# High-Resolution Morphological Approach to Analyse Elastic Laminae Injuries of the Ascending Aorta in a Murine Model of Marfan Syndrome

**DOI:** 10.1038/s41598-017-01620-8

**Published:** 2017-05-04

**Authors:** Júlia López-Guimet, Jordi Andilla, Pablo Loza-Alvarez, Gustavo Egea

**Affiliations:** 1 Departament de Biomedicina, Facultat de Medicina i Ciencies de la Salut, Universitat de Barcelona, Barcelona, Spain; 2 ICFO-Institut de Ciències Fotòniques, The Barcelona Institute of Science and Technology, 08860 Castelldefels, Barcelona Spain; 3 Institut de Recerca Biomèdica August Pi i Sunyer (IDIBAPS), Barcelona, Spain; 4 Institut de Nanociència i Nanotecnologia IN2UB, Universitat de Barcelona, Barcelona, Spain

## Abstract

In Marfan syndrome, the tunica media is disrupted, which leads to the formation of ascending aortic aneurysms. Marfan aortic samples are histologically characterized by the fragmentation of elastic laminae. However, conventional histological techniques using transverse sections provide limited information about the precise location, progression and 3D extension of the microstructural changes that occur in each lamina. We implemented a method using multiphoton excitation fluorescence microscopy and computational image processing, which provides high-resolution en-face images of segmented individual laminae from unstained whole aortic samples. We showed that internal elastic laminae and successive 2^nd^ laminae are injured to a different extent in murine Marfan aortae; in particular, the density and size of fenestrae changed. Moreover, microstructural injuries were concentrated in the aortic proximal and convex anatomical regions. Other parameters such as the waviness and thickness of each lamina remained unaltered. In conclusion, the method reported here is a useful, unique tool for en-face laminae microstructure assessment that can obtain quantitative three-dimensional information about vascular tissue. The application of this method to murine Marfan aortae clearly shows that the microstructural damage in elastic laminae is not equal throughout the thickness of the tunica media and in the different anatomical regions of the ascending aorta.

## Introduction

In mammals, the heart periodically ejects blood to the aorta, which is the main elastic artery in the body. The specific histological composition of the aorta allows an elastic response to blood ejection, which consists in the circumferential stretching of its wall and the subsequent recoil. The aortic wall is divided into three layers^1^: (i) the innermost layer named the tunica intima, composed of a monolayer of endothelial cells and subendothelial connective tissue that covers the luminal surface of the vessel; (ii) the tunica media, the thickest layer, is composed of elastic fibres arranged as fenestrated sheets (called elastic lamellae or laminae) alternating with circumferentially oriented layers of smooth muscle cells; and (iii) the tunica adventitia, the outermost layer, is composed of loose fibroelastic connective tissue enriched in collagen fibres and fibroblasts. Media elastic lamellae are concentrically arranged, with smooth muscle cells, collagen, proteoglycans and other extracellular matrix components filling the interlamellar space^2, 3^. The main function of these lamellae is to provide the elasticity needed for the aorta to stretch and recoil. The lamellae are wavy when the aorta is non-pressurized, and straight when subjected to *in vivo* blood pressure^4^. The most luminal lamina, named the internal elastic lamina (IEL), serves as a frontier between intimal endothelium and the tunica media. Transversely sectioned, conventional histological preparations of the aortic wall show elastic lamellae arranged in almost equidistant parallel layers, whose number depends on the animal species, and vessel calibre^5^, and is 7 to 8 on average for adult mice^6^. In addition, the surface of lamellae contains fenestrae, which are small holes of 1–10 µm in diameter^7^. Their size and density depend on the analysed vessel, animal species and age^8–10^. Their role is not yet well-established, but it is thought that they facilitate the flow of nutrients and the connection between cells located in different interlamellar spaces, and contribute to the developmental modelling of the IEL^11^.

It is of fundamental importance to preserve the integrity of all the aortic wall components in order to maintain effective vessel function^12^. In some pathologic conditions, the aortic structure is severely altered, which compromises its vital role in blood conduction. An example of this aberrant structure occurs in Marfan syndrome (MFS)^13^. MFS is an autosomal dominant heritable disorder that affects the cardiovascular, skeletal, ocular, pulmonary and nervous systems. MFS is caused by mutations in the fibrillin-1 gene (*Fbn1*)^14^, which encodes for fibrilin-1 protein, a basic component of the medial elastic lamellae. These mutations can directly affect the assembly of the matrix of the aortic media, leading to life-threatening aortic aneurysms^15^. Conventional histological studies show that Marfan aortic samples are characterized by fragmentation and disorganization of elastic fibres, accumulation of amorphous matrix components, fibrotic collagen production, and loss of cells^12, 13^. Moreover, the elastin content in human Marfan aorta is almost 50 per cent lower^12, 16^, and scanning electron microscopy demonstrates a significant loss of interlamellar fibres that link neighbouring laminae^17^. To study Marfan disease process, mice have been genetically engineered to replicate the clinical spectrum of the human disease. One of the most representative models is the *Fbn1* ^C1039G/+^ murine model, in which a cysteine is substituted with a glycine at amino acid 1039 in an EGF domain of the protein^18^, mimicking the most frequent type of mutation in human MFS patients. This Marfan murine model shows the formation of ascending aortic aneurysm, with the accompanying fragmentation of elastic fibres, overactivation of TGF-β signalling, disarray of smooth muscle cells, and disorganization of the collagen network^18, 19^.

The usual approach to examine the aforementioned aortic histological alterations is by conventional histological methods using fixed, dehydrated, paraffin-embedded, sectioned and stained samples. In recent decades, this approach has provided important knowledge about aneurysm initiation and progression in MFS and other aortopathies^20–22^. Nevertheless, the knowledge obtained using this methodology is inherently based on a two-dimensional perspective of the tissue, which is highly limiting to determine the location, progression and extension of the tunica media injuries when a three-dimensional (3D) microstructural analysis is required. For instance, it is not feasible to study fenestrae by classical histological methods due to their small size and distribution along the surface of lamellae. Therefore, for further insights, more sophisticated microscopy techniques have been developed and progressively applied to the cardiovascular system, including the aorta^23, 24^. In particular, multiphoton microscopy is well-suited for arterial wall imaging, since it permits visualization of almost the entire wall, without the need for exogenous fluorophores, or even sample fixation and embedding^25^. In the arterial case, multiphoton microscopy takes advantage of two nonlinear optical phenomena: two-photon excitation fluorescence (TPEF) or autofluorescence, and second harmonic generation (SHG)^26^. Based on endogenous tissue sources of nonlinear signals, the TPEF signal arises from the elastin content in elastic lamellae and the SHG signal originates from collagen fibres located at the adventitia and interlamellar spaces. Taking into account that these two matrix components make up most of the arterial tissue structure, multiphoton microscopy can disclose almost all the framework of an unstained aortic wall^25^. In addition, vascular samples can be imaged without tissue sectioning, using specific microscopy setups^27^. To date, some laboratories have used this technique to image aortic samples in a conventional transverse perspective (XZ or YZ axes)^25, 28^, as in standard histological preparations. A few other groups have applied multiphoton microscopy to image the tissue in an en-face view, and subsequently generated a three-dimensional rendering of it^29, 30^. The en-face histological perspective consists of visualizing the surface of the vessel (XY axes) along the depth of its wall. An illustrative example of en-face visualization is that obtained by endoscopic imaging. This unique view is very useful for the analysis of lamellae fenestrae, since they are localized on the laminae surface. Taking into account that conventional histological preparations of arterial tissue are sectioned in the transverse plane, fenestrae have historically been visualized by scanning electron microscopy^8^. However, this technique can only display the natural surface of the tissue, and hence studies have mainly focused on the IEL^7, 8^. Furthermore, the recent application of TPEF microscopy on rat artery samples has allowed the visualization and analysis of IEL fenestrae only^10, 31^, and has also been used in combination with exogenous fluorophores^32^. En-face TPEF microscopy has been used in Marfan mice to observe elastic lamellae fenestrae changes and thus report an elastolytic process, but with low-resolution imaging and very limited quantitative analysis^33, 34^. To our knowledge, no further data is available on lamellae 3D microstructure features in health or disease.

In this context, our aim was to provide further insights into the characterization of elastic lamellae microstructure, including fenestrae features, using the ascending aorta of MFS *Fbn1* ^C1039G/+^ mice as a histopathological model of tunica media alterations that typically occur in aortic aneurysms. To achieve this aim, we applied a recently implemented microscopy and computational method that we developed, which provides the en-face TPEF image of segmented individual lamellae from unstained whole aortic samples. The approach takes advantage of high resolution en-face multiphoton microscopy to characterize lamellae structural alterations in detail without sectioning the tissue. In addition, our study adds to the literature a semi-automatic image processing protocol that can isolate individual lamellae, which can be used to analyse microstructure in complex arterial wall samples. These two advantages allow the comparison of lamellae features within different anatomical regions of the vessel and within different lamellae from the same XY location. In particular, the application of our methodology to MFS murine aortae showed relevant lamellae fenestrae differences between IEL and 2^nd^ laminae, and between the proximal-concavity and the rest of the anatomical regions in the ascending aorta.

## Results

### New multiphoton en-face imaging and analysis of unstained aorta

**Figure 1:**
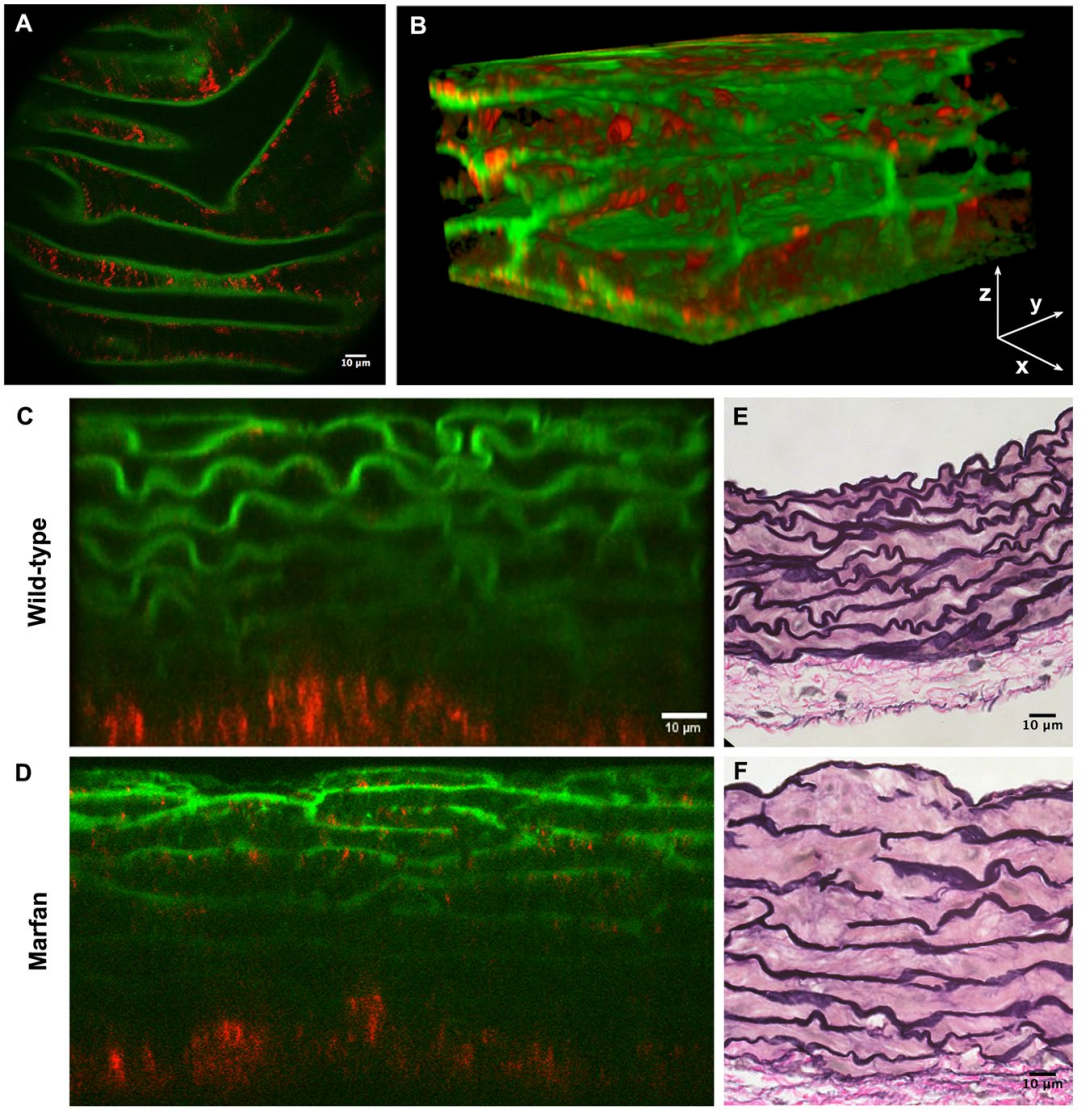
Aortic tissue visualizations using multiphoton microscopy. (**A**) En-face perspective of the IEL of the ascending aorta from a WT mouse using multiphoton microscopy. TPEF signal of elastin in green and SHG signal of collagen in red. (**B**) Three-dimensional rendering of a portion of the tunica media from the ascending aorta of a WT mouse. (**C** and **D**) Representative multiphoton images of the tunica media in transverse views of WT (**C**) and Marfan (**D**) mice. (**E** and **F**) Conventional histological visualization of elastic fibres using Verhoeff-van Gieson staining in WT (**E**) and Marfan (**F**) aortic tissue. Scale bar, 10 μm.

To obtain high-quality en-face images of intact mice aortae that allow a detailed assessment of lamellae microstructural alterations, we established a new method involving tissue preparation, image acquisition, processing, and analysis. To begin with, the entire aorta was dissected from the animal and immediately fixed in formol. Then, the vessel was cut in half longitudinally and mounted with the tunica intima facing onto the cover slide. This sample mounting arrangement allowed en-face imaging of the vessel surface as it would be seen by *in vivo* endoscopy. In addition, mounting the sample in this way was the most suitable approach to let microscopic light get through the vessel wall from the inner intima to the outermost adventitia layer, thus avoiding the expected premature light absorption by the presence of abundant adventitial collagen. Next, the ascending portion of aortic samples was imaged using a custom-made multimodal microscope^35^, which permitted simultaneous visualization of the elastic lamellae by elastin TPEF and collagen fibres by SHG. Thus, we could image the medial elastic lamellae (green) and the adventitial collagen plus the medial collagen fibres (red) (Fig. 1A–D). Acquisitions consisted of an en-face z-stack of confocal images, beginning at the tunica intima and running until the elastin signal became too low for the subsequent segmentation. Loss of signal was due to ordinary light absorption and scattering, and it was evident at ~60 μm inside the tissue (see the transverse views of 80 μm depth in Fig. 1C and D). Since acquisitions were taken en-face (XY axes) (Fig. 1A) and running into the tissue (Z axis), image stacks allowed visualization and understanding of the tissue in three dimensions (XYZ axis), and provided a vision of the entire volumetric structure of the aortic tunica media (Fig. 1B). Consequently, the transverse image of the aortic tissue can be obtained by visualizing the 3D image volume from the XZ or YZ perspectives (Fig. 1C and D), which was comparable with conventional histological preparations visualized by bright field microscopy (Fig. 1E and F).

**Figure 2:**
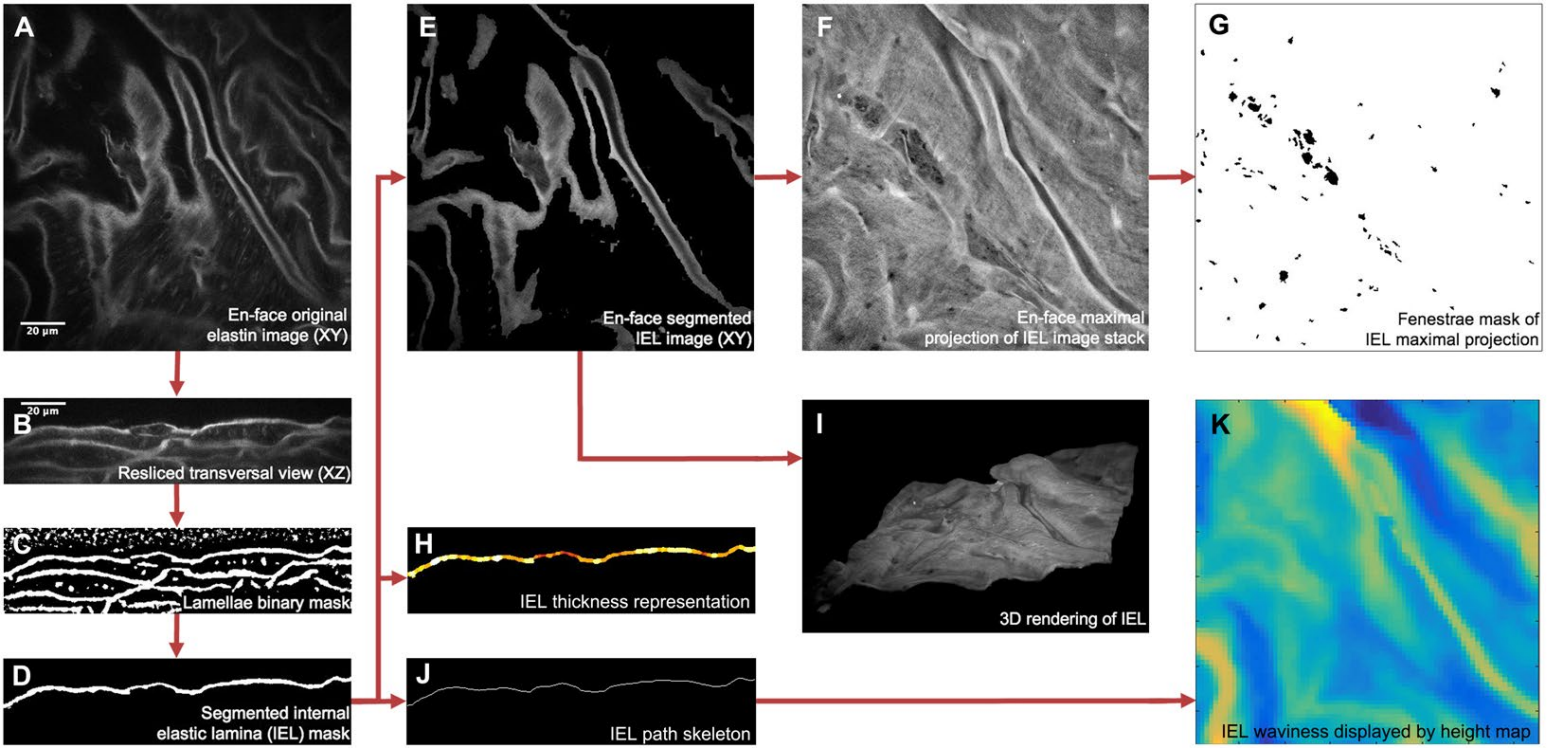
Image processing protocol to obtain en-face segmented elastic laminae and subsequent quantitative analysis. (**A**) Representative image of an acquired original en-face TPEF stack. Elastin TPEF and background cell autofluorescence signals are visualized in grey scale. (**B**) Representative image of the resliced original stack to transverse view. (**C**) Representative image of the binary mask stack that was subsequently obtained. Elastin signal and some background spots are automatically marked in white, the rest of the tissue is marked in black. (**D**) Representative image of segmented individual lamina mask stack. In this case, only the IEL mask was selected. (**E**) Representative image of the resulting en-face IEL stack. (**F**) En-face maximal projection comprised of all images within the IEL stack. (**G**) Binary mask of all fenestrae seen at the maximal projection (**F**). (**H**) Representative image of thickness display stack. Highest thickness is marked by white colour. (**I**) 3D rendering of the lamella obtained from the IEL stack. (**J**) Representative image of lamella skeleton image stack. (**K**) Height map showing global lamella waviness. Yellow denotes low and dark blue high heights. Scale bar, 20 µm.

The series of consecutive transverse images of elastin signal often showed the progression of lamella branching, small breaks, crosslinking between neighbouring lamellae and/or abrupt ending of lamellae (Supplementary Figure 1). These findings showed that aortic elastic lamellae are arranged in a 3D cage-like network^1, 2^, with irregularities that disrupt the apparent parallel arrangement seen in conventional histological preparations. Moreover, this view did not allow detailed lamellae microstructure visualization, and therefore segmentation of individual lamellae out of the acquired TPEF stack was then necessary. Consequently, to work with *clean* individual elastic lamella images, we developed a semi-automatic segmentation protocol in ImageJ software^36^ that processed the original elastin stack (Fig. 2). Briefly, each en-face image stack (XY) was virtually resliced to build its corresponding transverse image stack (YZ) (Fig. 2A and B), and then binary auto-thresholding was used to discriminate the elastin signal from the background (Fig. 2C). The chosen elastic lamella was then manually selected, and a new mask image stack was created with only this selection (Fig. 2D). The complete lamella mask stack was resliced back to the en-face view, and finally, it was applied to the original one (Fig. 2B) to generate the image stack of the selected individual elastic lamella (Fig. 2E). The en-face lamella stack provided 3D information on an isolated lamella, which could be represented as a 3D rendering (Fig. 2I) or a maximal projection image (Fig. 2F). Either of these representations revealed topological and structural data, but we chose the latter to perform a straightforward quantitative analysis. Thus, the surface of lamellae in two dimensions was displayed in its maximal projection image, so that its microstructure could be studied. Each fenestra (i.e. dark holes) found in the maximal projection of individual lamella was selected and marked in a fenestrae map (Fig. 2G). From this map, we carried out a morphological analysis to assess the density and size of fenestrae.

Our methodology could also be used to assess lamellae thickness, without any further procedure, by applying the BoneJ plugin for ImageJ^37^. This program quantifies lamella thickness and generates a visual colour representation from blue (lowest thickness) to white (highest thickness) (Fig. 2H).

Another lamellar feature that could be evaluated from the image acquisitions was the waviness of each lamella. To this aim, we developed a new approach based on measuring the height of the lamella in each pixel position of the image stack (Fig. 2J). This procedure, however, required a single Z axis height value per X-Y position. To this aim, we created a new protocol combining ImageJ and MATLAB software, which refined each segmented lamella image stack and its subsequent skeleton. Once the lamina skeleton had been obtained (Fig. 2J), we could evaluate its waviness in terms of height variation in the Z axis. Height values were organized into histograms and waviness maps represented by colours ranging from yellow (the lowest) to dark blue (the highest) (Fig. 2K).

### En-face microstructural analysis of wild-type and Marfan aortic elastic lamellae

Next, we applied the aforementioned methodology to examine in detail the elastic lamellae in the ascending aortic tissue in wild-type (WT, n = 4) and Marfan mice (MF; *Fbn1* ^C1039G/+^ model, n = 6). Four en-face z-stack acquisitions were randomly taken from each aorta sample, and fenestrae data were obtained from the maximal projection of each of the segmented lamellae. For each image stack, we segmented and analysed the IEL and the elastic lamina located just underneath it (2^nd^ lamina) to obtain the mean fenestrae density and the distribution of fenestrae sizes for each lamella maximal projection. We also measured lamellae thickness and waviness for each image stack. The transverse view of the aortic wall obtained by TPEF (Fig. 1C and D) closely matched what is seen in conventional histological preparations using Verhoeff-van Gieson staining (Fig. 1E and F). Marfan tissue visualized by both techniques showed lamellae disruptions and disarrangement (Fig. 1D and F) compared to WT tissue (Fig. 1C and E).

**Figure 3:**
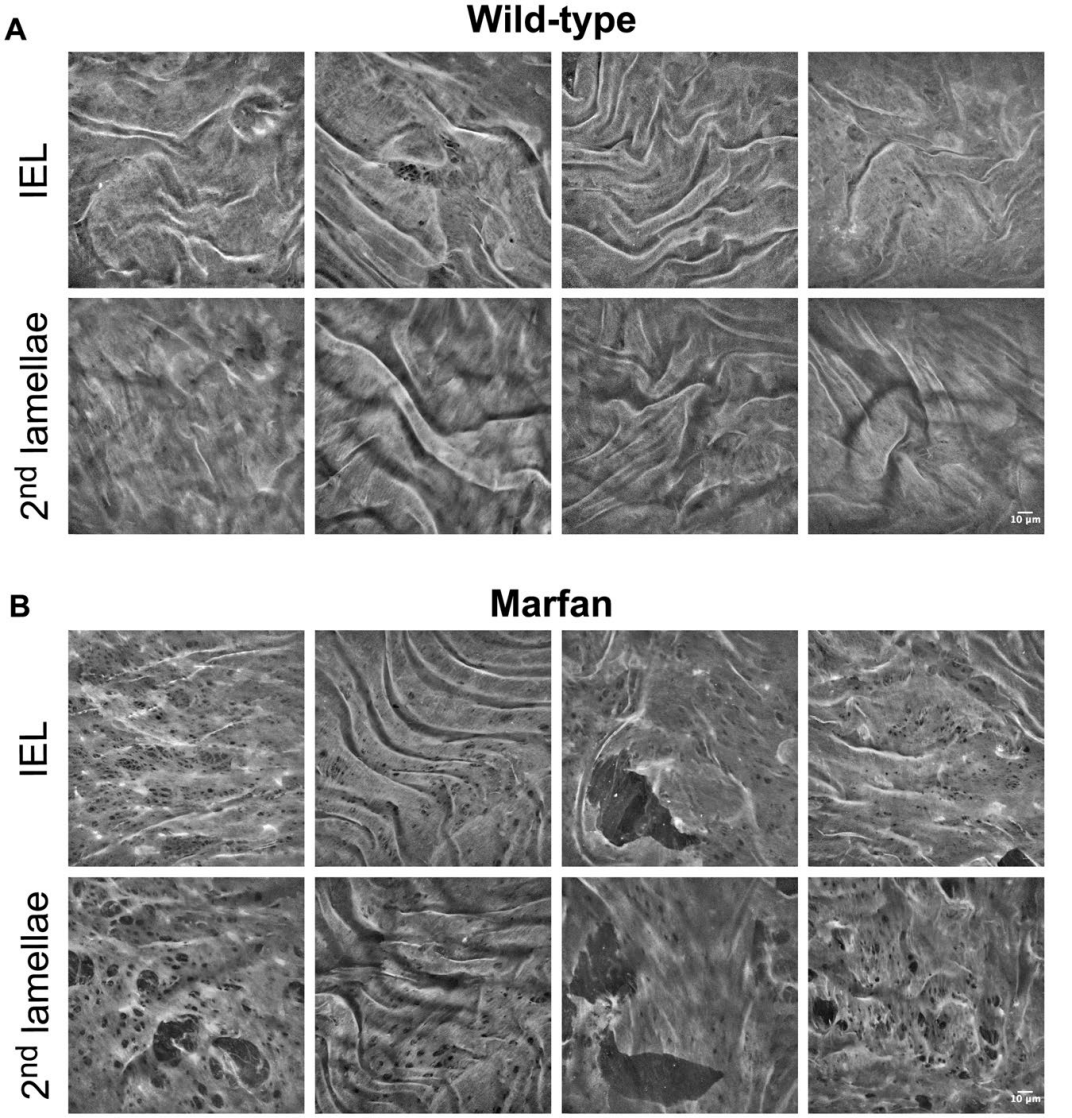
Representative en-face images of wild-type and Marfan elastic laminae. Maximal projections of segmented IEL and 2^nd^ lamina of WT (**A**) and Marfan (**B**) aortae. IEL and 2^nd^ laminae images of each column belong to the same image stack acquisition. In addition, each column corresponds to a different animal. Fenestrae are seen as black holes of variable size. Big polygonal black holes are considered ruptures, and are excluded from fenestrae quantification. Scale bar, 10 μm.

The en-face view showed that the IEL in WT animals had a flat, continuous aspect with unevenly distributed small fenestrae, which are visualized as small black holes (Fig. 3A). The IEL in Marfan mice showed more prominent fenestrae and occasional large ruptures (Fig. 3B). It could be postulated that these lamellar ruptures might be merely caused by sample handling during the surgery. To elucidate this, we measured the length of the rupture’s hole at different points in the XY maximal projection. Our aim was to mimic the quantitation of the length for elastic laminae breaks when aortae are examined transversally in classic histological preparations (Fig. 1F). Thus, to resemble conventional histological sectioning, we used a standard grid that marked all the horizontal and vertical lines where length measurements should be performed for all ruptures. This way, the maximal projection rupture length was on average 20.71 µm at IEL and 30.73 µm at the 2^nd^ lamellae. Altogether, the length of Marfan ruptures was 26.52 µm (±19.29 µm). For comparative reasons, we quantified the length of elastic laminae breaks from 24 conventional histological preparations of Marfan aortae (Fig. 1F), giving a mean length of 20.95 µm (±19.47 µm). Note that the values obtained from en-face and transversal histology are very similar and therefore, the ruptures in Marfan en-face images of IEL and 2^nd^ lamellae (Fig. 3B, third column panels from the left) correspond to the classical elastic laminae breaks observed by conventional histological methods and not primarily caused by sample handling.

**Figure 4:**
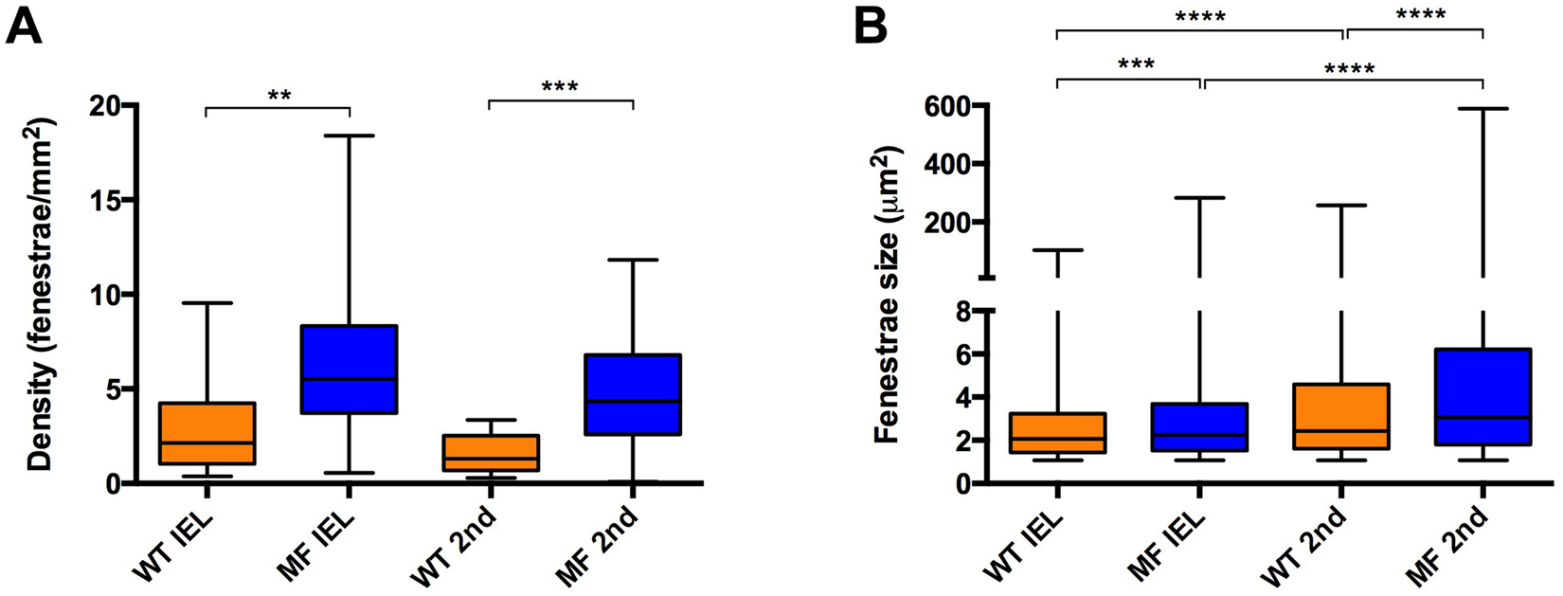
Quantitative analysis of density and size of fenestrae seen in IEL and 2^nd^ elastic laminae from en-face maximal projection images. Fenestrae density (**A**) and size (**B**) seen in WT (orange) and Marfan (MF, blue) IEL and 2^nd^ lamellae. Statistical significance between groups is indicated by asterisks, and defined in the Materials and Methods section. Interquartile boxplots with minimum and maximum whiskers. Forty maximal projections were analysed and a total of 6,400 fenestrae were quantified.

Despite having an identical genetic background, Marfan mice showed variable lamina aspect patterns. However, the differences with WT mice were clearly evident. In particular, fenestrae density was 2.5 fold higher in Marfan IEL than in WT IEL (Fig. 4A; median values: 2.14 fenestrae/mm^2^ in WT vs. 5.53 fenestrae/mm^2^ in MF). Marfan mice also had significantly larger fenestrae than WT mice (Fig. 4B; 2.07 µm^2^ in WT vs. 2.25 µm^2^ in MF). Consequently, the total area of elastic lamina occupied by fenestrae in the field of view was clearly larger in Marfan mice.

The 2^nd^ elastic laminae were segmented and analysed in the same way as the IEL. We observed that the structural differences between WT and Marfan 2^nd^ laminae were even more evident than in the IEL (Fig. 3). The results of quantitative analysis of the 2^nd^ laminae were very similar to those obtained in the IEL when we compared WT and Marfan mice. Fenestrae density and size were significantly greater in the 2^nd^ lamellae of Marfan mice than in WT mice (Fig. 4; density: 1.29 fenestrae/mm^2^ WT vs. 4.34 fenestrae/mm^2^ in MF; size: 2.43 µm^2^ WT vs. 3.06 µm^2^ in MF). No differences in fenestrae density were observed between the IEL and 2^nd^ lamellae in WT and Marfan mice (Fig. 4A). However, there was a significant increase in the size of fenestrae between IEL and the 2^nd^ lamellae in WT and Marfan aortae (Fig. 4B), which was more pronounced in Marfan aortae (WT: 2.07 µm^2^ in IEL vs. 2.43 µm^2^ in 2^nd^ lamina; MF: 2.25 µm^2^ in IEL vs. 3.06 µm^2^ in 2^nd^ lamina).

**Figure 5:**
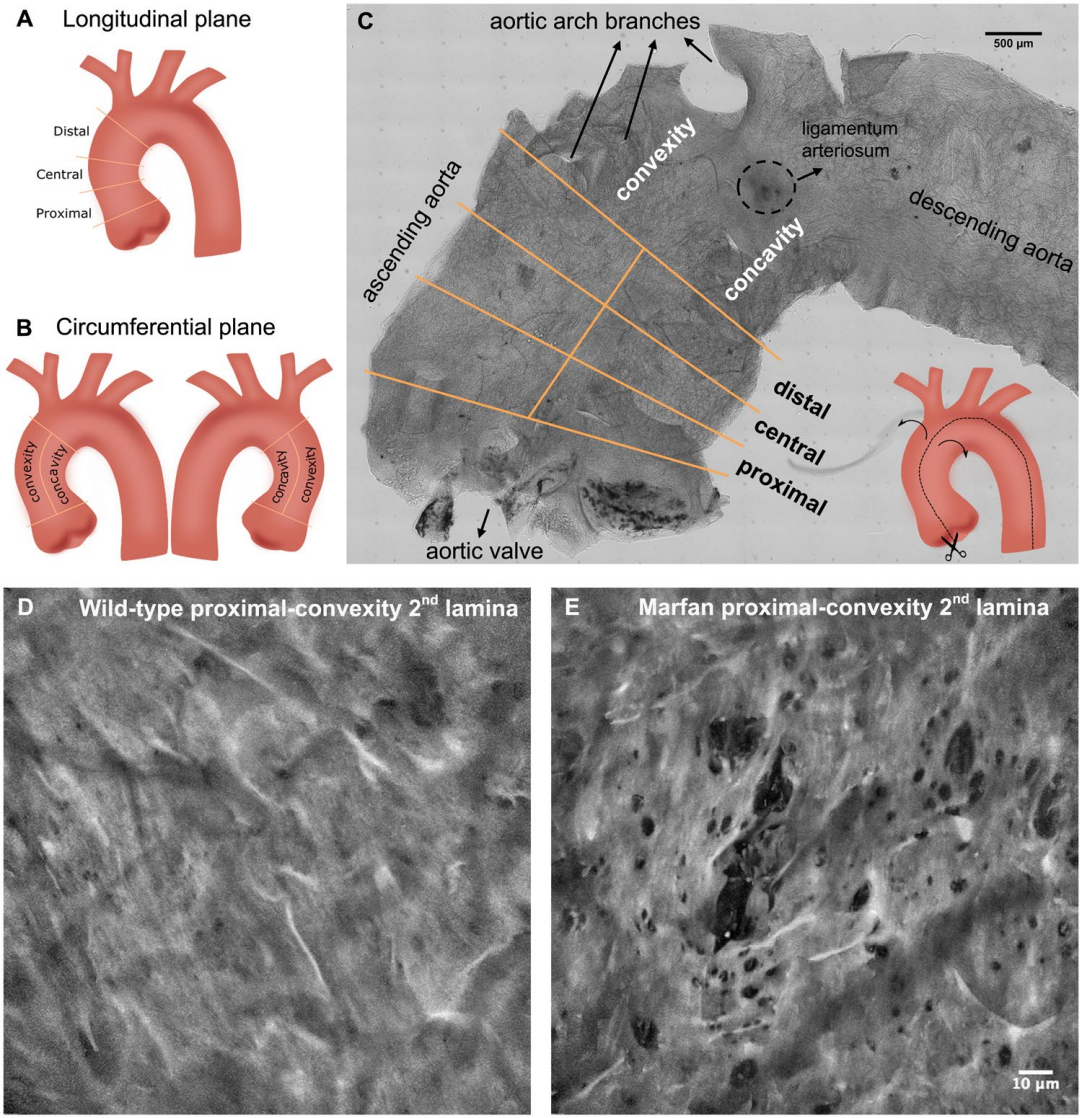
Anatomical regionalization of ascending aorta used in this study. (**A** and **B**) Schematic drawings of defined ascending aorta anatomical regions used in our study in longitudinal (**A**) and circumferential (**B**) planes. (**C**) Bright field image map of the longitudinally open aorta (as shown in the inset schematic drawing) and the corresponding regions. (**D** and **E**) Representative maximal projections acquired in the proximal-convex region of WT (**D**) and Marfan (**E**) aortae. Scale 500 μm in (**C**) and10 μm in (**D**,**E**).

**Figure 6:**
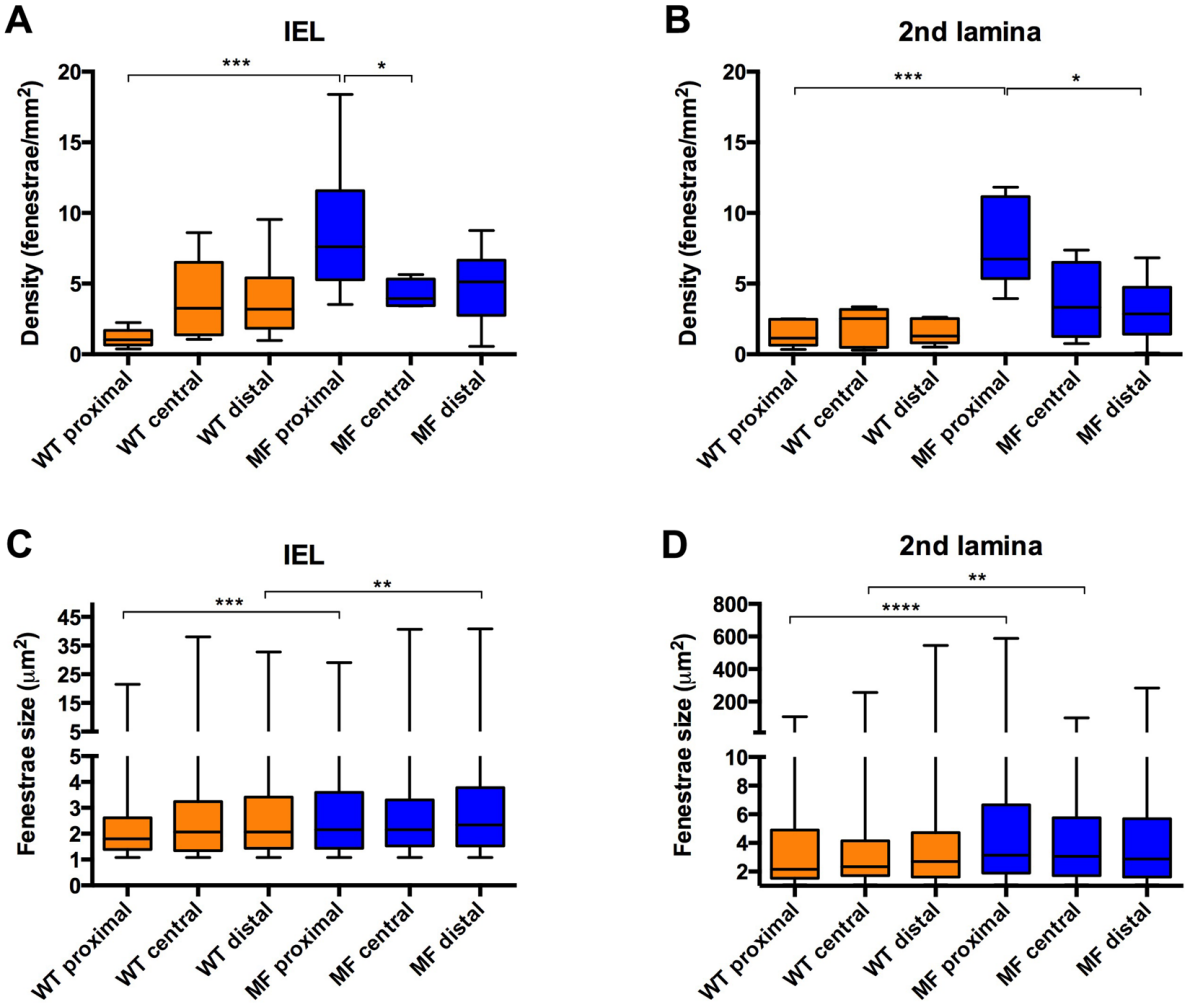
Quantitative analysis of fenestrae in IEL and 2^nd^ laminae in the longitudinal plane of the ascending aorta. The density of fenestrae in the IEL (**A**) and 2^nd^ lamina (**B**), and their respective individual fenestrae size (**C** and **D**) measured at different longitudinal plane locations (proximal, central and distal) of WT (orange) and Marfan (MF, blue) ascending aortae. Statistical significance between groups is indicated by asterisks. Interquartile boxplots with minimum and maximum whiskers. Forty maximal projections were analysed and a total of 6,400 fenestrae were quantified.

Next, we performed a systematic analysis of the density and size of fenestrae in different anatomical locations of the ascending aorta. The locations were defined as proximal, central and distal in the longitudinal plane, and as concavity and convexity in the circumferential plane (Fig. 5A–C). A qualitative examination of images of the aforementioned regions revealed that laminae obtained in the convexity and proximal zones apparently had more microstructural damage (Fig. 5D and E). Indeed, statistically significant differences were observed between WT and Marfan mice in the IEL and in the 2^nd^ elastic lamina, mainly in the proximal and convex regions. In particular, the median density of fenestrae in proximally located IEL (Fig. 6A) and 2^nd^ laminae (Fig. 6B) were 7.5 and 6 times higher respectively in Marfan than in WT mice (IEL: 1.02 fenestrae/mm^2^ WT vs. 7.60 fenestrae/mm^2^ in MF; 2^nd^ lamella: 1.14 fenestrae/mm^2^ in WT vs. 6.74 fenestrae/mm^2^ in MF). Moreover, Marfan fenestrae were significantly larger at proximal and distal IEL (Fig. 6C), and at proximal and central 2^nd^ laminae (Fig. 6D).

**Figure 7:**
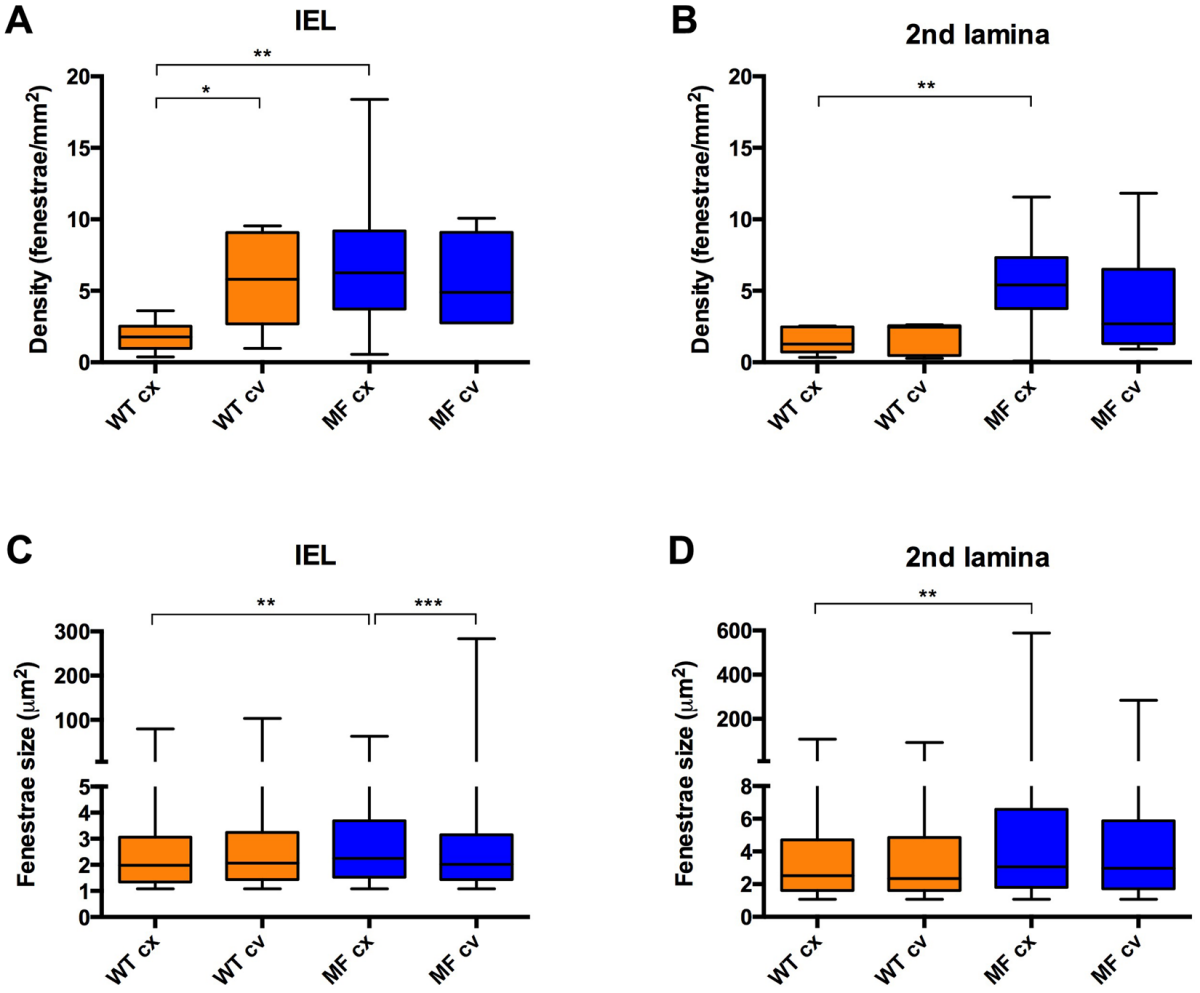
Quantitative analysis of fenestrae in IEL and 2^nd^ laminae in the circumferential plane of the ascending aorta. The density of fenestrae at the IEL (**A**) and 2^nd^ lamina (**B**), and their respective individual fenestrae size (**C** and **D**) measured at different circumferential plane locations (convexity/cx and concavity/cv) of WT (orange) and Marfan (MF, blue) ascending aortae. Statistical significance between groups is indicated by asterisks. Interquartile boxplots with minimum and maximum whiskers. Forty maximal projections were analysed and a total of 6,400 fenestrae were quantified.

The circumferential partition of data (Fig. 7) showed that fenestrae density in WT IEL located at the concavity (cv) was significantly higher than that at the convexity (cx) of the aorta (Fig. 7A; 1.78 fenestrae/mm^2^ in cx vs. 5.81 fenestrae/mm^2^ in cv). However, this was not the case for WT 2^nd^ laminae (Fig. 7B; 1.27 fenestrae/mm^2^ in cx, and 2.46 fenestrae/mm^2^ in cv). In contrast, Marfan IEL fenestrae density ranges at concavity and convexity (2.8–9.1 fenestrae/mm^2^) were highly similar to that at WT concavity (2.7–9 fenestrae/mm^2^), and greater than at WT convexity (1–2.5 fenestrae/mm^2^) (Fig. 7A). At the 2^nd^ laminae, Marfan fenestrae density was similar to that at the Marfan IEL (compare MFcx with MFcv in Fig. 7A and B), and higher than 2^nd^ laminae WT density in both circumferential regions (Fig. 7B), but was only significant at the convex region. We observed a significant difference in the size of fenestrae between Marfan and WT convexities both at the IEL and the 2^nd^ laminae (Fig. 7C and D, respectively; IEL: 1.98 µm^2^ in WT vs. 2.25 µm^2^ in MF; 2^nd^ laminae: 2.52 µm^2^ in WT vs. 3.06 µm^2^ in MF). In summary, structural injuries in the Marfan ascending aortae were regionalized, and were preferentially accumulated in the convexity region of the circumferential plane, and mainly in the proximal region of the longitudinal plane.

Finally, we also examined potential differences between WT and Marfan mice regarding lamellae thickness and waviness. Lamellae thickness showed that in WT and Marfan, IEL and 2^nd^ lamellae were on average 2.7–3.0 µm, without significant differences between them (Supplementary Figure 2A). In addition, the measurement of waviness showed that WT and Marfan lamellae had the same spectrum of height values (Supplementary Figure 2B).

## Discussion

In this article, we report the implementation of a new multiphoton microscopy image processing method for elastic lamellae microstructure examination, based on obtaining series of en-face images from unstained aortic tissue. We used healthy and MFS murine aortae as tissue models and determined the anatomical distribution of fenestrae alterations that occur in elastic laminae. In the last decade, a lot of data have been generated about histological damage in the aortic wall in MFS, using routine histological techniques following the conventional sequence of paraformaldehyde/formol fixation, paraffin embedding, sectioning and histological (immune) staining. Technological improvements in microscopy and importantly in image processing have provided a new panorama to the histopathology field^2^. Accordingly, here we applied en-face multiphoton microscopy and a segmentation protocol to assess lamellae morphology. The advantages of our new approach are: (i) it can produce high-resolution en-face confocal stacks that enable detailed visualization of histological structures; (ii) it can obtain quantitative information belonging to the three dimensions XYZ, which increases our understanding of 3D histological arrangements; and (iii) the entire aortic vessel can be viewed, for straightforward monitoring of different anatomical regions.

Although TPEF confocal microscopy has been used to obtain the elastin signal of vessels in other studies^25^, our report is the first to our knowledge in which a semi-automatic image processing protocol is systematically implemented to segment individual lamellae and quantitatively analyse histological microstructural changes. Furthermore, this unique analysis was performed on en-face images of ascending aorta tunica media of a murine model of MFS, in which elastic fibre ruptures are known to be associated with the formation and progression of aortic aneurysm^18^. Related previous studies in MFS reported “disruptions” in en-face images from pressurized adult MF mice descending aortae, but without any quantitative analysis^33^; and also reported a ≈40 μm “hole” diameter measurement based on non-segmented lamellae of diseased tissues only (from another MFS murine model)^34^. In addition, the measurement of elastic laminae features of transversely viewed parts of Marfan mice aortae obtained by multiphoton microscopy was reported as an alternative to conventional histological methods^38^. Here, we complement these studies and highlight the relevance of en-face multiphoton microscopy and image processing for generating quantitative 3D microstructural data on individual elastic lamellae. We report new histopathological alterations in the aortic media in the murine *Fbn1* ^C1039G/+^ model of MFS: lamellae in the ascending aorta show larger and more fenestrae than WT tissue. The density of fenestrae in Marfan elastic laminae is at least double that found in WT. These fenestrae alterations probably represent lamellar micro-damage, which could be directly related to the characteristic elastic lamellae fragmentation and disarrangement happening in Marfan aortae^38^.

Interestingly, our results show that alterations in the density and size of fenestrae did not occur uniformly in the entire Marfan ascending aortic media, but were mostly restricted to the proximal and convex regions. This is in accordance with results reported by ref. 39 in the ascending aorta of angiotensin II-infused ApoE^−/−^ mice. Using conventional histological techniques, they reported that the largest aortic wall dissections occurred in the outer convex quadrant, which corresponds to the central part of the aortic convexity. We speculate that the regionalized structural changes reported here could be due to a preferential impact of the blood flow on the convex and proximal ascending aortic regions. It is known that deviant blood flow can be caused by aortic valve dysfunction^40^. In particular, the convexity of ascending aorta is the preferential blood flow impact zone found in bicuspid aortic valve disease^41^, which is accompanied by differential lamellae fragmentation and matrix protein expression patterns in comparison to the concavity^42^. In the case of MFS patients, aortic root dilatation usually entails aortic valve dysfunction^43^, which in turn causes aortic blood flow disturbance^43, 44^ leading to mild or moderate aortic regurgitation^44, 45^. Therefore, it is reasonable to hypothesize that MFS disturbed flow could be mainly impacting on this particular anatomical zone, just as happens in bicuspid aortic valve pathology. This premise should be confirmed by further detailed hemodynamic studies on Marfan patients and murine models, which to our knowledge are not currently available.

A recent paper^39^ reported a transmural gradient of lamellar injury, in which elastic laminae break number varies significantly amongst lamellae. In particular, central lamellae including the 2^nd^ showed more breaks than peripheral ones. The results of our comparisons of fenestrae density and size between the IEL and the 2^nd^ lamina also suggest a histological injury gradient, where the damage is more severe in the 2^nd^ lamina than in the IEL. Again, we can only speculate about the significance of this difference. It could be related to intrinsic robustness of the IEL, whose structure and organization might be better adapted to support the mechanical impact of blood flow than the rest of laminae. In this respect, aortic transmural mechanical behaviour has been studied in relation to transmural structural properties^46, 47^. Results show that the porcine thoracic descending aorta wall is divided into transverse outer and inner tunica media halves, which differ in their mechanical and molecular composition. In addition, the alignment of bovine elastic and collagen fibres due to mechanical load varies in lamellae localized close to the endothelium and in the subsequent lamellae^48^. We are aware that this variance cannot be directly extrapolated to mice, due to differences in the animal model used and the aortic portion examined in terms of wall thickness and lamellae number. Nonetheless, it cannot be discarded that similar structural variance between lamellae occurs in mice as well. Our results suggest that this variance might take place between IEL and successive lamellae. In the case of Marfan mice aortae, we hypothesise that the weakness of the tunica media^16, 49^ plus the intrinsically different primary structure of each lamina could explain the here reported dissimilar injury pattern occurring between the 2^nd^ laminae and the IEL.

Regarding the other lamellar parameters assessed by our methodology, it was previously shown by SEM that medial elastic laminae of adult rat aorta were 2–3 µm thick and had an irregular profile^50^. Moreover, an average of 2.74 µm was reported in mouse^51^. Therefore, our lamella thickness values (2.7–3.0 µm) are in accordance with those measured in mouse^51^ and rat aortae^50, 52^.

There are few data about lamellae undulation assessment either in health or disease. Wolinsky and Glagov^4^ established the waviness index, which consisted in obtaining the ratio of the lamellar length to the straight line distance between two reference points. Other developed undulation assessment techniques quantified the folding^53^ and fiber angular undulation^28^ of lamellae. Globally, these three methods are all based on individual transversal sections, and hence they provide 2D data. Conversely, our waviness quantification approach takes into account area values as in earth topography studies, and therefore it is much more informative about the 3D structure of the tissue. With this method, we showed no differences in lamellar waviness between WT and Marfan aortic tissue.

In conclusion, here we describe the method that we have developed, apply it to wild-type and Marfan murine aortae, and quantify morphological differences in terms of lamellae fenestrae, thickness and waviness features. Our results take advantage of multiphoton microscopy to achieve en-face images of unstained aortic tissue, which in turn provide us with novel information on lamellae 3D histopathological damage. The application of this methodology to Marfan mice aneurysm-prone tissue suggested the density of fenestrae as a potential aortic microscale damage marker, whose alterations are mainly accumulated in the proximal and convex regions of the ascending aorta. Finally, our method opens the door to study in detail 3D vessel morphology and injuries in other conditions, diseases, and animal models. The future application of our imaging and processing method as a basis for the vascular endoscopy examination of MSF patients or related diseases might provide an early evaluation tool for aortic histological damage prior to the irreversible appearance of the aneurysm.

## Methods

### Experimental animals and sample preparation

Nine-month-old *Fbn1* ^C1039/+^ mice and age-matched wild-type littermates were used in this study (n = 4 for WT and n = 6 for Marfan mice). Animal care and experimental procedures were approved by the University of Barcelona’s independent Committee for Animal Welfare, according to the University of Barcelona’s guidelines and the European Parliament Directive. The mice were on a C57B/6 genetic background and maintained as a heterozygous breeding colony in our animal room facility. Animals were sacrificed by isoflurane inhalation and the aorta was surgically harvested from the aortic root until its suprarenal portion, and immediately rinsed in PBS and fixed in formol 10% overnight. Thereafter, aortae were cut longitudinally (see inset in Fig. 5C). The open aorta was placed on a glass slide covered with mowiol, and some small transverse cuts were performed to keep the tissue flat. Each aorta was mounted with the tunica intima facing the coverslide. For this study, only the ascending aorta was used for imaging. A detailed map image of the entire sample was obtained by mosaic stitching of 308 bright field images of 0.8 × 0.5 mm field of view (see Fig. 5C). Bright field images were acquired using a 10 × 0.5 NA objective (Nikon) and Qimaging fast camera, with 0.46 μm pixel size.

### Multiphoton microscope setup and image acquisition

The microscope setup consisted of a custom-made non-linear optics setting, based on a fully motorized Ti eclipse Nikon microscope. Multiphoton excitation was obtained using a Coherent mira900 titanium sapphire laser. The laser produced pulses of ~150 fs with a repartition rate of 76 MHz, and the power used at the back aperture of the objective was 40 mW. To perform aorta imaging, the laser wavelength was set to 810 nm, producing at 405 nm a generation of second harmonic and efficient TPEF signals. The filters were the following: Semrock FF720-SDi01-25 × 36 for TPEF/SHG generation; Semrock FF01750/SP-25 for TPEF detection; Semrock FF735-Di01-25 × 36 for SHG detection; and Semrock FF01-406/15-25 for forward SHG detection. Samples were visualized using a 40 × 1.3 NA oil objective (Nikon) and the collection of the forward second harmonic signal was performed by means of a 1.4 NA oil immersion condenser. Image stacks of both signals (TPEF/elastin and SHG/collagen) were taken simultaneously by custom-made acquisition software coded in Labview. Image stacks were carried out at z-step 0.5 μm from the intima until 60 μm deep into the tissue. Acquisitions were taken at a pixel size of 0.29 μm, field of view 512 × 512 px and averaged 5 times. Four image stacks at different anatomical locations were acquired for each aorta sample and the exact location was assessed using the bright field image map of each sample.

### Image processing

Quantitative data was obtained by an image processing protocol (as indicated in Fig. 2) scripted in ImageJ macro language, available on demand. The elastin signal image stack was resliced to its YZ perspective, and automatically binary thresholded using the Niblack algorithm at radius 10 to generate an elastin binary mask. The resulting mask stack was separated into groups of 15 consecutive images (34 groups), and each group was manually processed to select the chosen elastic lamella and isolate it from the other lamellae. This segmented lamella mask stack was re-resliced to recover the XY perspective, and small errors of segmentation and thresholding were corrected by applying a binary erode routine of 50 iterations at range 5. Manual rectification of segmentation inaccuracies was executed when needed. The corrected lamella mask was applied to the original elastin image stack to obtain the isolated segmented lamella, and its maximal projection was created. Illumination intensity variations were adjusted. Sauvola local threshold at radius 5 was applied to the adjusted maximal projection image to create a fenestrae binary mask. Manual check and correction were performed to obtain a verified fenestrae dataset. From the fenestrae binary mask, individual area (μm^2^) and density (fenestrae number/mm^2^) of fenestrae greater than 1 μm^2^ were measured for each lamella using the “analyse particles” algorithm. The number of elastic lamellae ruptures and their area were excluded from the analysis. The image processing protocol yielded maximal projections of 16 wild-type IEL, 16 wild-type 2^nd^ lamellae, 24 Marfan IEL and 24 Marfan 2^nd^ lamellae. A total of 6,400 fenestrae at all maximal projections were quantified.

BoneJ plugin in ImageJ was used to automatically quantify lamella thickness from the segmented lamella stacks. For an accurate measurement, image stack voxel size was rescaled by 0.6 at the X and Y axis, so that voxels had isotropic dimensions (0.5 × 0.5 × 0.5 µm). Then the BoneJ specific measurement of thickness was applied onto each segmented lamella stack mask. Data was the mean thickness ±SD of the whole stack, and the programme provided a coloured representation of local thicknesses in each stack slice.

To quantify lamella waviness in ImageJ, the YZ segmented lamella mask stack was manually checked for any error in the continuity of the lamella. Next, 9 automated series of binary erode and dilate were applied to the lamella mask in order to smooth the mask surface without losing its path. The YZ mask stack was then converted into its skeleton (by the “Skeletonize” ImageJ algorithm), depicted as a black background and white single-pixeled line tracing the core path of the lamella and branches, in each one of the stack slices. The next step was to process the skeleton stack to erase all undesired branching. We developed a complex automated algorithm run on MATLAB (The MathWorks Inc., Natick, MA) that tracked each one of the white pixels in the skeleton and classified them into skeleton segments belonging to the lamellar core path or to a branch path. Once the classification had been done in each stack slice, the algorithm joined only the core path skeleton segments to finally generate the clean lamella skeleton stack. However, due to simplification of the 3D lamella shape into a split 2D skeleton, the lamellar smooth continuity was somehow spoiled. Therefore, in ImageJ, 9 series of erode and dilate were used automatically, first in the XY perspective and then in the resulting YZ view. Finally, a complete clean accurate skeleton stack was obtained from each segmented lamella stack. Out of these, we applied another developed MATLAB algorithm to generate height data. The algorithm tracked each white pixel in the YZ skeleton to get its height value in the Z axis, and relativized each value to the minimum height value of the whole skeleton. To eliminate noise or tiny details affecting the wave pattern, the XY image (made of the relativized height values) was rescaled by 0.15. Likewise, a possible general inclination of the lamella was also corrected from the relativized rescaled height image by subtracting its own 20px Gaussian blurred image. At the end, these final height values were displayed as a height map where yellow denotes low heights and dark blue represents high ones.

### Conventional histology

Formol-fixed mice aortae were dehydrated and embedded in paraffin. Five µm transverse sections were stained with Verhoeff-Van Gieson staining for visualization of elastic fibres. The length of elastic laminae breaks was measured manually from 24 images, using ImageJ. The mean break length and standard deviation were calculated.

En-face lamellar rupture length corresponded to the rupture distance crossed by a vertical or horizontal line. To standardize, a 10 × 10 µm grid was superimposed on all the TPEF maximal projection images that showed lamellar ruptures (8 out of the 48 total Marfan images). Each rupture yielded multiple length values. The mean length of ruptures was calculated by averaging the multiple lengths obtained from all the ruptured images. The standard deviation was also calculated in order to show data dispersion. Ruptured images were relatively infrequent, distributed among half of the Marfan aortic samples, and their anatomical location was aleatory.

### Statistical method

Data were analysed using GraphPad Prism 6, and plotted as median and interquartile boxplots with minimum and maximum whiskers. As the datasets presented diverse distribution shapes, statistical analysis was carried out using the Kolmogorov-Smirnov nonparametric test. The value of P ≤ 0.05 was considered statistically significant. The degree of significance was assigned as follows: *for P ≤ 0.05, **for P ≤ 0.01, ***for P ≤ 0.001, and ****for P ≤ 0.0001.

### Data Availability

The datasets and protocols generated during the current study are available from the corresponding author upon request.

## Electronic supplementary material

video 1

Supplementary data
