# Supplementary material for: High-Resolution Morphological Approach to Analyse Elastic Laminae Injuries of the Ascending Aorta in a Murine Model of Marfan Syndrome
Source: Sci Rep. 2017 May 4;7:1505. doi: 10.1038/s41598-017-01620-8 (PMC5431420; doi:10.1038/s41598-017-01620-8)
Supplement: Supplementary file 2 — Supplementary data [file 41598_2017_1620_MOESM2_ESM.pdf]

SUPPLEMENTARY INFORMATION

High-Resolution Morphological Approach to Analyse Elastic Laminae Injuries of the Ascending Aorta in a Murine Model of Marfan Syndrome

Júlia López-Guimet[1], Jordi Andilla[2], Pablo Loza-Alvarez[2], Gustavo Egea[1,3,*]

[1]Departament de Biomedicina, Facultat de Medicina, Universitat de Barcelona, Barcelona (Spain)

[2]ICFO-Institut de Ciències Fotòniques, The Barcelona Institute of Science and Technology, 08860 Castelldefels, Barcelona, Spain.

[3]Institut de Recerca Biomèdica August Pi i Sunyer (IDIBAPS), Barcelona (Spain)

Lopez-Guimet et al-supplementary video.avi

Supplementary figure 1 (video). **Consecutive image sequence of elastin signal at transverse view**. Resliced original elastin image stack from wild-type aortic tissue, where lighter grey signal displays elastic lamellae. Progression throughout the volume shows lamellae branching, crosslinking, and breaks. Stack tissue volume dimensions are 153.6x66.0x153.6 µm (XZY).

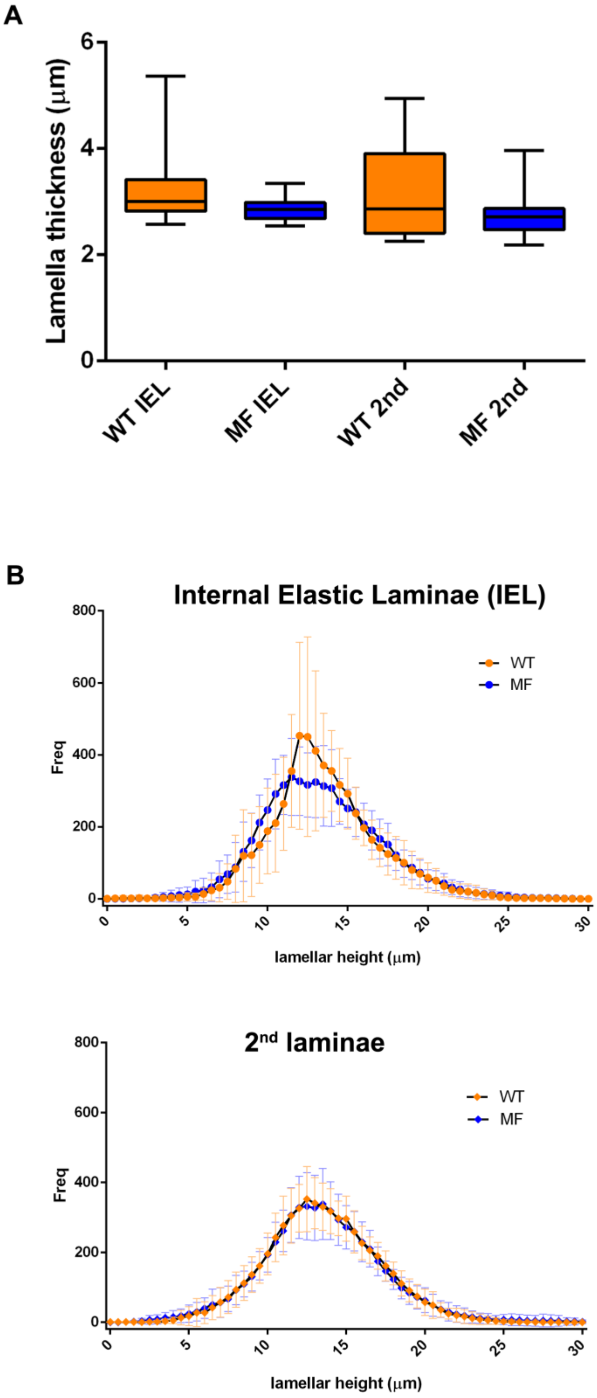

Supplementary figure 2. **Quantitative analysis of lamella thickness and waviness**. (A) Thickness in wild-type (orange) and Marfan (blue) IEL and 2nd laminae. (B) Waviness by lamella local height histogram.
